# Genetics of Base Coat Colour Variations and Coat Colour-Patterns of the South African Nguni Cattle Investigated Using High-Density SNP Genotypes

**DOI:** 10.3389/fgene.2022.832702

**Published:** 2022-06-07

**Authors:** Langelihle Mbali Kunene, Farai Catherine Muchadeyi, Khanyisile Hadebe, Gábor Mészáros, Johann Sölkner, Trevor Dugmore, Edgar Farai Dzomba

**Affiliations:** ^1^ Discipline of Genetics, School of Life Sciences, University of KwaZulu-Natal, Scottsville, South Africa; ^2^ Agricultural Research Council, Biotechnology Platform, Onderstepoort, South Africa; ^3^ Division of Livestock Sciences, University of Natural Resources and Life Sciences, Vienna, Austria; ^4^ KZN Department of Agriculture and Rural Development, Pietermaritzburg, South Africa

**Keywords:** genome-wide association study, nguni cattle, coat colour, colour-sidedness, white forehead stripe

## Abstract

Nguni cattle are a Sanga type breed with mixed *B. taurus* and *B. indicus* ancestry and proven resistance to ticks, diseases and other harsh conditions of the African geographical landscape. The multi-coloured Nguni coats have found a niche market in the leather industry leading to breeding objectives towards the promotion of such diversity. However, there is limited studies on the genomic architecture underlying the coat colour and patterns hampering any potential breeding and improvement of such trait. This study investigated the genetics of base coat colour, colour-sidedness and the white forehead stripe in Nguni cattle using coat colour phenotyped Nguni cattle and Illumina Bovine HD (770K) genotypes. Base coat colour phenotypes were categorised into eumelanin (*n* = 45) and pheomelanin (*n* = 19). Animals were categorised into either colour-sided (*n* = 46) or non-colour-sided (*n* = 94) and similarly into presence (*n* = 15) or absence (*n* = 67) of white forehead stripe. Genome-wide association tests were conducted using 622,103 quality controlled SNPs and the Efficient Mixed Model Association eXpedited method (EMMAX) implemented in Golden Helix SNP Variation Suite. The genome-wide association studies for base coat colour (eumelanin vs. pheomelanin) resulted into four indicative SNPs on BTA18 and a well-known gene, MC1R, was observed within 1 MB from the indicative SNPs (p < 0.00001) and found to play a role in the melanogenesis (core pathway for melanin production) and the MAPK signalling pathway. GWAS for colour-sidedness resulted in four indicative SNPs, none of which were in close proximity to the KIT candidate gene known for colour-sidedness. GWAS for the white forehead stripe resulted in 17 indicative SNPs on BTA6. Four genes MAPK10, EFNA5, PPP2R3C and PAK1 were found to be associated with the white forehead stripe and were part of the MAPK, adrenergic and Wnt signalling pathways that are synergistically associated with the synthesis of melanin. Overall, our results prove prior knowledge of the role of MC1R in base coat colours in cattle and suggested a different genetic mechanism for forehead stripe phenotypes in Nguni cattle.

## Introduction

The Nguni cattle are a Sanga breed native to South Africa (Makina et al., 2014) and farmed in other countries worldwide. This breed is of great importance to South Africa due to its ability to survive under very harsh climatic conditions coupled with ticks and tick borne diseases (Mapiye et al., 2007). Nguni cattle are thought to have survived through strong selection during their migration from North Africa to Southern Africa, leading to phenotypic changes in the animals and making them capable of withstanding the harsh environments of the country (Bester et al., 2003). Traits displayed by the Nguni cattle in order to survive in extreme climate with limited feed and exposure to tick infestations are relevant to resource limited smallholder farmers (Bester et al., 2003) who rely on Nguni cattle for food security (milk and meat) and by-products (hides, skin and horns) that they use to generate income (Musemwa et al., 2010). Nguni cattle have diverse coat colours with different patterns, which, in the previous years, led to the belief that they were admixed with other breeds and were thus considered inferior to exotic breeds of uniform and standard coat colour patterns (Bester et al., 2003). Three main coat colours exist in Nguni cattle which are black, brown and red and the other coat colours that are found in the breed are modifications of these (Olson 1999).

The unique and diverse Nguni cattle coat colours are of cultural significance particularly to the Nguni ethnic people (Oosthuizen 1996). Because of their cultural values, there is a strong bond between the Nguni cattle and Nguni people leading to instances such as poetic naming of the animals of different physical attributes with inspiration arising from the beautiful coat colour and patterns on their hides (Oosthuizen 1996). For example, the, white Nguni cattle with black spots reminds the Nguni people of flies in the buttermilk and is named “Inasenezimbukane” while black cattle with a white pattern resembling a spitting cobra are named “iFezi” (Abundant Herd, 2003). Black cattle for instance are used when a king is deceased and during celebrations such as rain ceremonies (Oosthuizen 1996). In addition to the socio-cultural roles, Nguni cattle hides have an economic value in the manufacturing of leather products for the automotive, furniture and fashion industries (Brits 2014). Cattle hides are exported overseas for car upholstery and are locally used for the manufacture of furniture, handbags, horse-riding equipment and clothing garments (footwear and belts) (Brits, 2014). Coupled to these socio-cultural and economic roles, coat colour in livestock including cattle is also important because as it has been observed to be associated with other traits of economic importance (Brits 2014). Different coat colours in Nguni cattle are associated with resistance and susceptibility to pests/tick infestation and diseases that can result in unwanted culling or death of certain animals (Mapholi et al., 2014). Because of these socio-cultural, economic and adaptive associations, farmers across commercial and smallholder sectors have been observed to have preferences for certain coat colour patterns. In a study on trait preferences for smallholder Nguni bulls, coat colour was the sixth trait of interest after aggression and mating behaviour, tick and disease resistance, body condition score, scrotal circumference and body size and conformation (Tada et al., 2013). Coat colour was also ranked as the fifth preferred trait after tick and disease resistance, reproductive efficiency, body condition score and body size and conformation in Nguni cows (Tada et al., 2013). In order to inform selection and breed improvement programs, understanding the genetics of coat colour becomes a prerequisite.

The mechanism of coat colouration (melanogenesis) is determined by the melanin found within the melanosomes in the melanocytes (Marks and Seabra 2001) and can either be categorised into either eumelanin and pheomelanin (Olson 1999). Eumelanin is entirely responsible for black to brown pigmentation of the skin and coat colours while pheomelanin is responsible for red to yellow colouration of mammal coat colours (Olson 1999). The production of eumelanin relies on the stimulation of a G-protein coupled receptor called melanocyte-specific melanocortin receptor (MC1R) by agonists α-melanocyte-stimulating hormone (α-MSH) and adrenocorticotropic hormone (ACTH) (Millington 2006). The antagonist of the MC1R, agouti signalling protein (ASIP) controls the production of eumelanin by stimulating pheomelanogenesis (Videira et al., 2013). Melanogenesis is a process where melanin is synthesized in the presence of an amino acid called tyrosine (TYR). Mutations of the TYR amino acid results in unpigmented coat colours and the mutation is termed albinism (Cieslak et al., 2011). When melanogenesis is disrupted, leucism can occur. Leucism is a condition where there is lack of pigmentation due to the absence of melanocytes on the animals’ skin and/or coat and results in what is seen as white spots or patches on a solid base colour. These phenotypes include the white forehead stripe on the cattle’s heads and colour-sidedness patterns as observed in the Texas Longhorn, Florida Cracker, English Longhorn, Scandinavian cattle and other African breeds (Olson 1999). Mutations of the KIT and KITL genes have been reported to have association with white spotting and roan phenotypes in Belgian Blue and Shorthorn cattle (Seitz et al., 1999). However, other studies such as that conducted by Hofstetter et al. (2019) reported BTA22, which harbour the MITF gene to be associated with the white phenotype in Brown Swiss cattle. According to Bennett and Lamoreux (2003), a large number of genes affects coat colour and colour patterns through different mechanisms (Bennett and Lamoreux 2003). According to Fontanesi et al. (2010) genetic heterogeneity is prevalent in coat colour phenotypes in cattle and other livestock species.

The Illumina HD bovine Beadchip (Illumina, Inc., San Diego, CA, United States) was developed by a multi-institutional consortium, Bovine HapMap, and contains 777,962 SNP markers. The taurine, zebu and composite breeds were used in the development of the array (Harris et al., 2011) and the chip has found utility in population genetics (Meseret et al., 2020), genomic evaluation and selection (Uemoto et al., 2015), copy number variation (Jiang et al., 2013) and case-control genome-wide association studies (GWAS) (Riffert 2015). Riffert (2015) observed the MC1R gene to be associated with the dark coat colours of the Brazilian Gir cattle. Colour-sidedness was reported to be a result of the copy number variant (CNV) mechanism on a 480 kb region that harbours the KIT gene on BTA6 in cattle (Durkin et al., 2012). The Illumina BovineHD Beadchip was used to investigate and identify markers for slick hair coat on three tropical breeds, Senepol, Carora and Romosinuano, the study of which reported S-phase kinase-associated protein 2 (SKP2) and sperm flagellar 2 (SPEF2) as candidate genes for the slick hair phenotypes in these cattle breeds (Huson et al., 2014). The KIT gene was also associated with white base colour and spotting legs of Gir cattle (Riffert 2015). GWAS on facial markings have also been explored in the Fleckvieh cattle and the results showed that the MITF on BTA22 and KITLG on BTA5 were found to be associated with pigmentation patterns on the animal’s head (Mészáros et al., 2015).

Despite its socio-cultural and economic importance, very little is known about the genetics of coat colour in Nguni cattle, absence of this information of which hinder efforts for genetic improvement for such traits. The aim of this study was therefore to investigate genomic regions (SNPs and candidate genes) and genetic mechanisms that are associated with the coat colours and patterns in South African Nguni cattle using the Illumina Bovine HD genotypes and Nguni cattle phenotyped for base coat colour, colour-sidedness and presence and absence of white forehead stripes. The Nguni cattle used in this study were from two research populations of Bartlow Combine and Kokstadt in KwaZulu-Natal province and consisted of unrelated animals representative of the Nguni cattle diversity.

## Materials and Methods

### Animal Populations and Coat Colour Phenotypes

**FIGURE 1:**
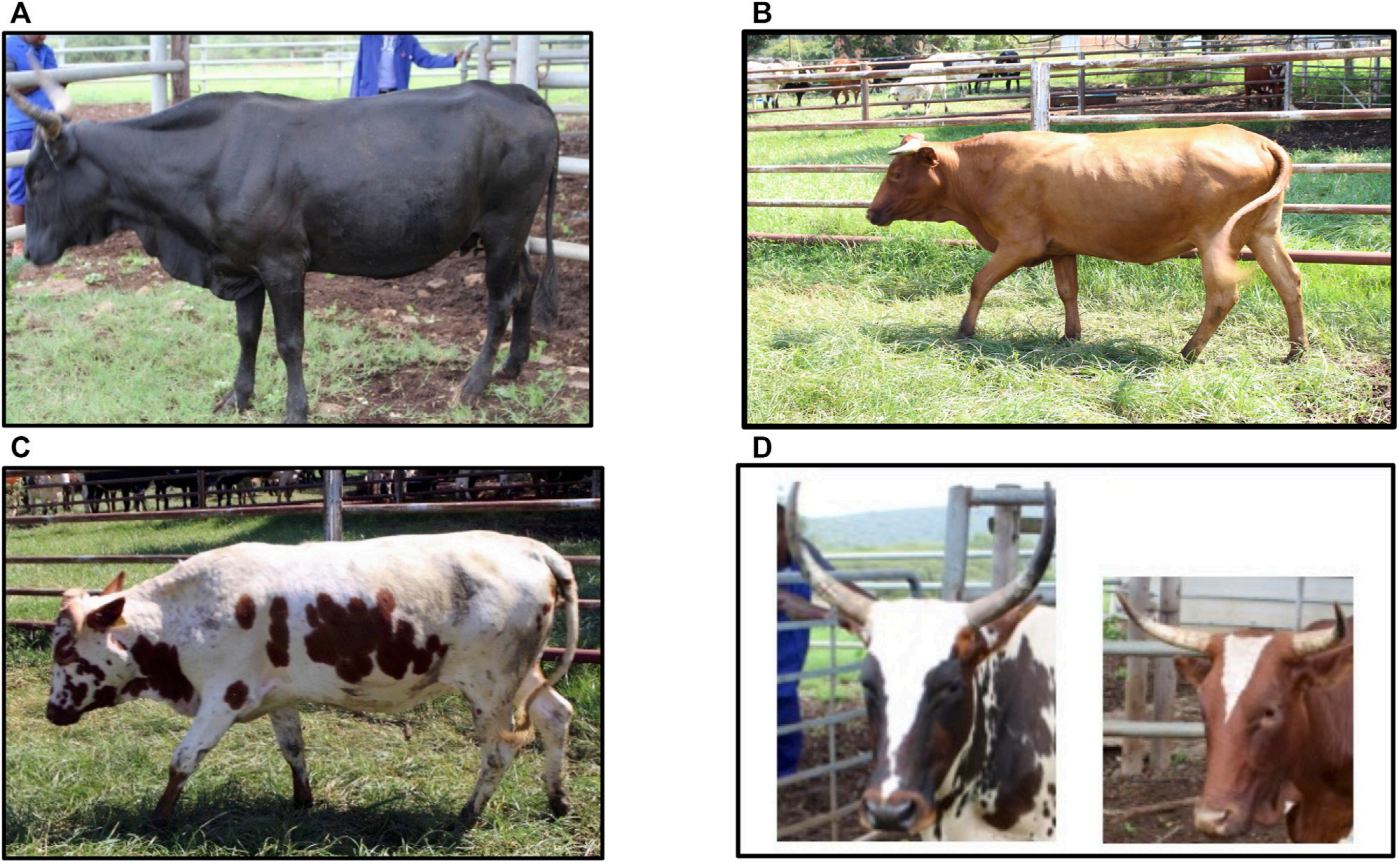
Nguni cattle depicting base coat colours and the colour-sidedness and the white forehead stripe pattern. **(A)** Black (eumelanin) coat coloured Nguni cattle, **(B)** Red (pheomelanin) coat coloured Nguni cattle, **(C)** A colour-sided Nguni cattle, **(D)** white forehead stripe observed in Nguni cattle.

A total of 142 Nguni cattle were sampled from two research stations of the Bartlow Combine (*n* = 78) and Kokstadt (*n* = 66) in the KwaZulu-Natal region. The Bartlow Combine and Kokstadt research stations are located at the North and Southern parts of KwaZulu-Natal province of South Africa respectively. These two research stations are conservation farms for the Nguni cattle diversity and were chosen as a representative sample of the total Nguni cattle diversity. Using photo images, the coat colour patterns were phenotyped into 1) black, brown or red base coat colours and 2) colour-sidedness and the 3) white forehead stripe patterns as illustrated in Figure 1. Base coat colour was classified into two different categories of 1) eumelanin which constituted black (*n* = 39) and brown (*n* = 6) animals and 2) pheomelanin with the red (*n* = 19) animals. Of the 64 cattle used in the base colour analysis, 35 were from the Bartlow Combine station and 29 were from the Kokstadt station. Forty-six (46) colour-sided and 94 non-colour-sided Nguni cattle were phenotyped and used in the colour-sidedness trait analysis. A total of 82 Nguni cattle were phenotyped for the white forehead stripe trait of which 15 animals had the white forehead stripe and 67 did not have the forehead stripe.

### Genotyping and Quality Control

Genomic DNA was extracted from the 142 blood samples using the QIAGEN MagAttract HMW DNA Kit (Qiagen Hilden, Germany). The concentration of the extracted DNA was measured using the qubit fluorometer (Life Technologies, United Kingdom) and concentrations greater than 25 ng/μL were accepted for further analysis. DNA integrity was verified on a 1% agarose gel. High-quality DNA was genotyped using the Illumina Bovine HD BeadChip Array kit (Illumina, Inc., San Diego, CA, United States) and in accordance with the Infinium HD Assay Super Manual Protocol at the Agricultural Research Council Biotechnology Platform, South Africa.

SNP quality control was conducted using PLINK version 1.9 (Chang et al., 2015). A genotyping rate for individual animals’ assessment was set at 0.95. SNPs were pruned based on a genotyping call rate of 0.95, Hardy-Weinberg < 0.00001, minor allele frequency <0.05. In addition, SNPs without chromosomal positions were removed from the data. The call rate of 0.95 removed 2 animals and 140 animals remained for downstream analysis. The assessment of SNP quality removed 8,463 SNPs for the genotyping call rate, 4,259 SNPs for Hardy-Weinberg and 142,290 SNPs for minor allele frequency. SNPs without chromosomal position (*n* = 847) were also removed and 622,103 SNPs remained for downstream analysis.

### Within-Population Genetic Diversity and Population Structure Analysis

Genetic variation within populations was estimated using four measurements of minor allele frequency (MAF), observed heterozygosity (H_O_), expected heterozygosity (H_E_) and inbreeding coefficients (F_IS_) all of which were analysed using PLINK version 1.9. Genetic relationships between the Nguni cattle from the Bartlow Combine and Kokstadt conservation stations were also analysed using Principal Component Analysis (PCA) and genetic relationship matrix (GRM) on Golden Helix SNP Variation Suite (SVS) version 8.5.0 (Golden Helix, Bozeman, MT, United States). PCA and GRM were also conducted for the animals within each coat colour phenotype (Base coat colour, Forehead stripe and colour sidedness) in order to investigate the genetic relationships between cases and controls for each GWAS.

### Genome-wide Association Analysis

Genome-wide association tests for the base coat colours and patterns were done using Golden Helix SVS version 8.5.0 (Golden Helix, Bozeman, MT, United States) using the Efficient Mixed Model Association eXpedited analysis (EMMAX) (Kang et al., 2010). The eumelanin was considered the case compared to pheomelanin; colour-sided animals were considered the case against non-colour-sided animals; and the animals with a white forehead stripe were regarded as the case against those without a forehead stripe, as illustrated in Table 1. The mixed model used in this study was:

y=Xα +Kμ+e

where *y* was the coat colour type or pattern, X was the genotype (622,103 SNP genotypes), *α* was regarded as the vector of the fixed effect (genotype) while K was the relative kinship matrix, μ was regarded as the unknown random effect, and e was the random error. The Bonferroni correction threshold for multiple tests was used to detect the genome-wide significant SNPs, which were defined as *α*/K (*α* = 0.05 and K is the number of SNPs = 622,103). Manhattan plots were generated using the Bonferroni adjusted log of the *p*-values on Golden Helix SNP Variation Suite (SVS) version 8.5.0 (Golden Helix, Bozeman, MT, United States). Per marker pairwise F_ST_ analysis was conducted using Golden Helix SNP Variation Suite (SVS) version 8.5.0 (Golden Helix, Bozeman, MT, United States) to investigate loci highly differentiating cases from controls.

**TABLE 1:** Case-control GWAS tests used for the generation of Manhattan plots.

Trait	# Case	# Control
Base coat colour	Eumelanin (39 Black, 6 Brown)	Pheomelanin (19 Red)
Colour-sidedness	46 Colour-sided	94 Non-colour-sided
White forehead stripe	15 with the white forehead stripe	67 without the forehead stripe

### LD and Population Stratification

In order to guide gene annotation, pair-wise LD (*r* ^2^) values were estimated between adjacent SNPs for each autosome and the genome-wide LD over all autosomes in each Nguni herd using Plink v1.09 (Purcell et al., 2007). LD decay LD decay was then analyzed for three maximum distances between SNP pairs, ≥10 kb, ≥100 kb, and ≥1,000 and for each distance, SNP comparisons were binned applying bin sizes of 1 kb, 10 kb, and 100 kb, respectively. The average *r* ^2^ for each bin was estimated and plotted against the inter-marker distance.

Furthermore, a quantile-quantile (Q-Q) plot was generated using Golden Helix SNP Variation Suite (SVS) version 8.5.0 (Golden Helix, Bozeman, MT, United States) in order to assess the influence of population stratification on the GWAS. In addition the average genomic inflation factors (*λ*), defined as the median of the resulting chi-squared test statistics divided by the expected median of the chi-squared distribution drawn from the EMMAX analysis.

### SNP Annotation

Candidate genes in close proximity (upto 1 MB downstream or upstream from the significant SNPs (p < 0.00001) were identified using the Golden Helix SVS GenomeBrowse using the BTAU5.0.1 (November 2015) reference genome. Pathways for the identified genes ±1 MB from the indicative SNP for each trait (base coat colour, colour-sidedness and white forehead stripe) were obtained from the Kyoto Encyclopedia of Genes and Genomes (KEGG) database and their association with coat colour patterns inferred.

## Results

### Genetic Diversity

Minor allele frequencies of 0.260 ± 0.137 and 0.265 ± 0.135 were reported for Bartlow and Kokstadt herds respectively (Table 2). The observed (H_O_) and expected (H_E_) heterozygosity for the Bartlow cattle were 0.361 ± 0.148 and 0.348 ± 0.135 respectively, which were similar to those observed in Kokstadt cattle (H_O_ = 0.365 ± 0.145, H_E_ = 0.353 ± 0.131). The observed heterozygosity for Bartlow Combine and Kokstadt populations were greater than the expected heterozygosity (H_O_ > H_E_), indicating the possibility of previously isolated populations coming together to form a single population. As a result, slightly negative inbreeding coefficients were observed for both Bartlow (−0.038 ± 0.037) and Kokstadt (−0.033 ± 0.060) cattle.

**TABLE 2:** Minor allele frequency (MAF), observed (H_O*)*_ and expected (H_E*)*_ heterozygosities and, inbreeding coefficient (F_IS) of Nguni cattle from_ Bartlow and Kokstadt herds.

Herd	N*	MAF ±SD	H_O_ ± SD	H_E_ ± SD	F_IS_ ± SD
Bartlow	73	0.260 ± 0.137	0.361 ± 0.148	0.348 ± 0.135	-0.038 ± 0.037
Kokstadt	67	0.265 ± 0.135	0.365 ± 0.145	0.353 ± 0.131	-0.033 ± 0.060

N = number of animals.

### Population Structure

**FIGURE 2:**
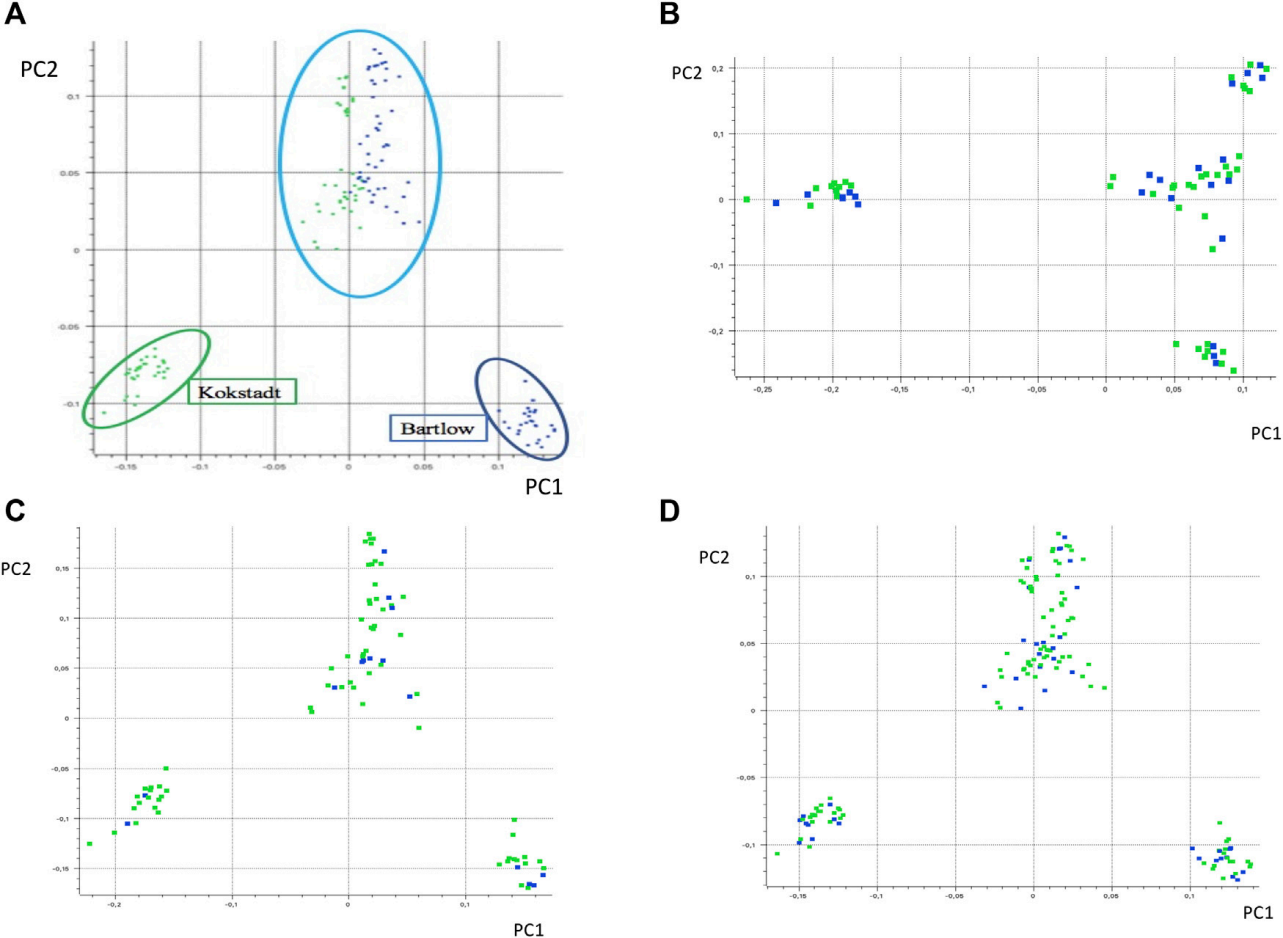
Principal component based clustering of the Nguni cattle **(A)** from two different farms, Bartlow Combine and Kokstadt conservation stations; **(B)** phenotyped for base coat colourl **(C)** phenotyped for absence and presence of forehead strip and **(D)** phenotyped for colour sidedness or non-colour sidedness. Principal component analysis diagram showing principal component one (PC1) and principal component two (PC2) and the variance depicted alongside the axes of the diagram.

**FIGURE 3:**
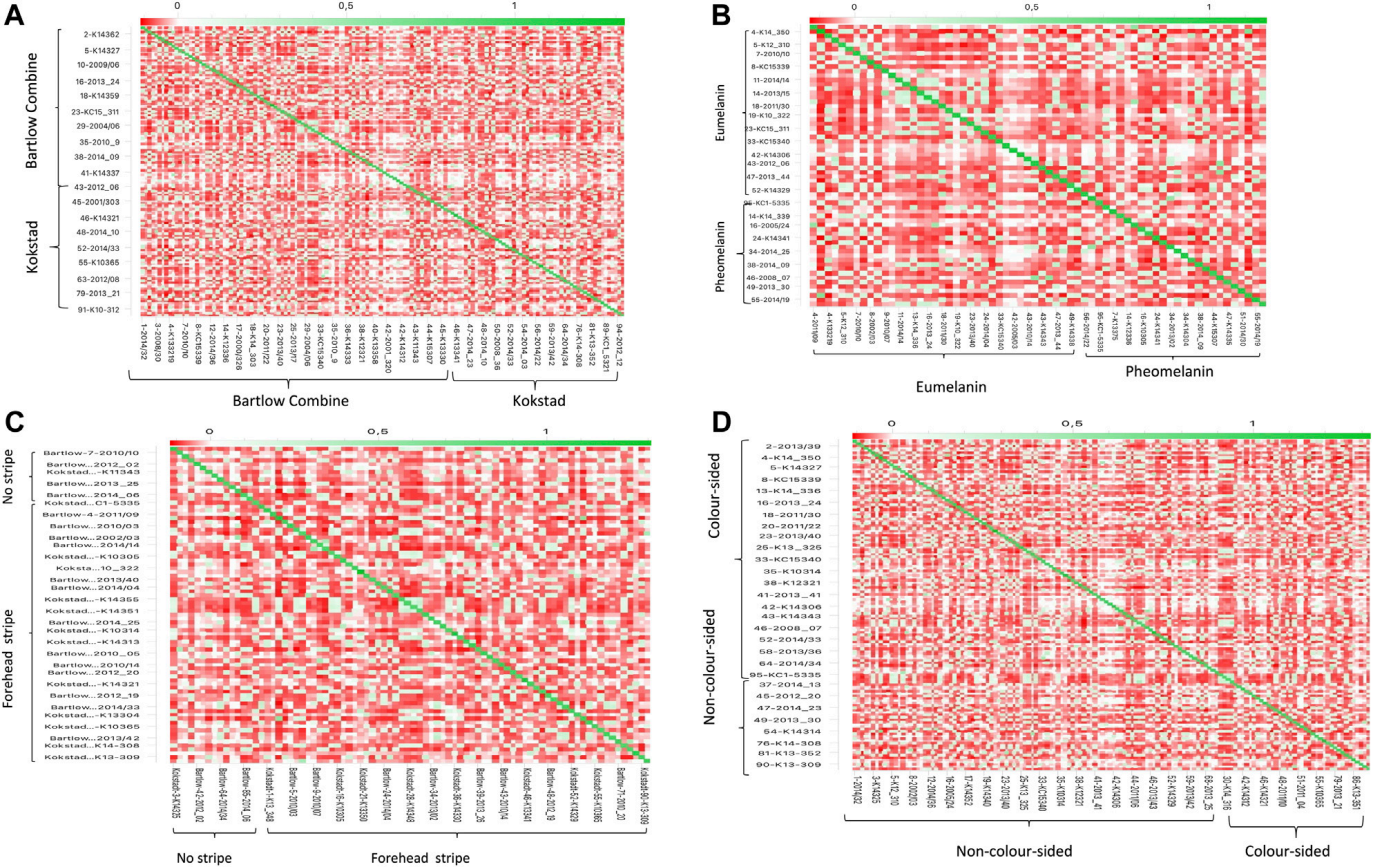
Heat Map of the genomic relationship matrix for **(A)** Bartlow Combine and Kokstadt conservation stations; **(B)** Eumelanin and pheomelanin Nguni cattle **(C)** Nguni cattle with or without forehead stripes and **(D)** colour sidedness and non-colour sided Nguni cattle. The animal identities (IDs) are shown on the *x* and *y*-axis.

PC1 and PC2 together accounted for 40% of the total variance, and separated the Nguni cattle into three different clusters (Figure 2A). PC1 (21.1% of the variation) separated the Nguni cattle of Kokstadt station (green) from those of Bartlow Combine (blue). PC2 (18.8% of the variation) separated the two clusters of Cattle (Kokstadt and Bartlow combine) from a single cluster that was constituted by animals from the two research stations. Population structure, analysed using PCA analysis explained 11, 13 and 18% of the genetic variation in the animals phenotyped for base coat colour, colour sidedness and Forehead stripe respectively (Figures 2B–D). In all three coat colour phenotypes, PC1 and PC2 explained less than 40% of the total genetic variation and animals randomly clustered into three groups with no clear distinction of cases and controls (Figures 2B–D). The genetic relationship matrix (GRM) heatmap (Figure 3) showed the kinship within and between herds with estimates ranging from 0 (low kinship) to 1 (high kinship). Low Kinship was observed in both herds of Bartlow (0.037 ± 0.013) and Kokstad (0.043 ± 0.013) and Relatively lower (-4.46 × 10^–12^ ± 0.013) kinship was observed between pairs of animals from the two herds (Figure 3A). Similarly low kinship was observed within and between cases and controls of base coat colour (1.47 × 10^–13^), presence and absence of forehead stripe (4.05 × 10^–15^) and colour sided (2.02 × 10^–12^) phenotypes respectively (Figures 3B–D).

### LD Analysis and Population Stratification

An average genomewide LD (r2) of 0.413 ± 0.219 for Bartlow and 0.402 ± 0.209 for Kokstad was observed. LD decay analysis demonstrated that LD stayed just above 0.2 upto 600 kb and decayed to just about 0.1 at 1000 kb window (Supplementary Table S1).

**FIGURE 4:**
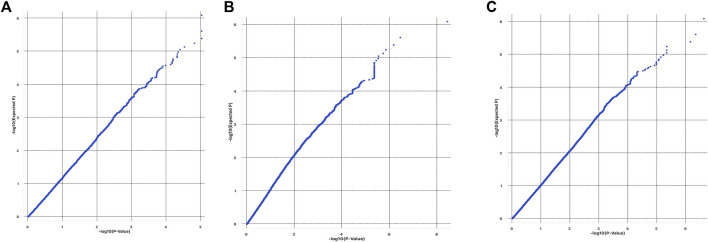
QQplots from GWAS of **(A)** base coat colour; **(B)** Forehead stripe and **(C)** colour sided phenotypes.

The quantile-quantile plots (Q-Q plots) show that the influence of population stratification was negligible (Figures 4A–C). Moreover, the average genomic inflation factors (*λ*) for the three phenotypes were close to 1 (1.03 for Base coat colour; 0.96 for colour sidedness and 1.09 for Forehead stripe). Both the QQ plots and *λ* suggested little or no residual population structure effects on the test statistic inflation. Despite the small sample size, the results of GWAS are therefore reasonable and worth further investigation.

### Genome-wide Association: SNPs, Genes and Pathways

#### Base Coat Colour (Eumelanin vs. Pheomelanin)

**FIGURE 5:**
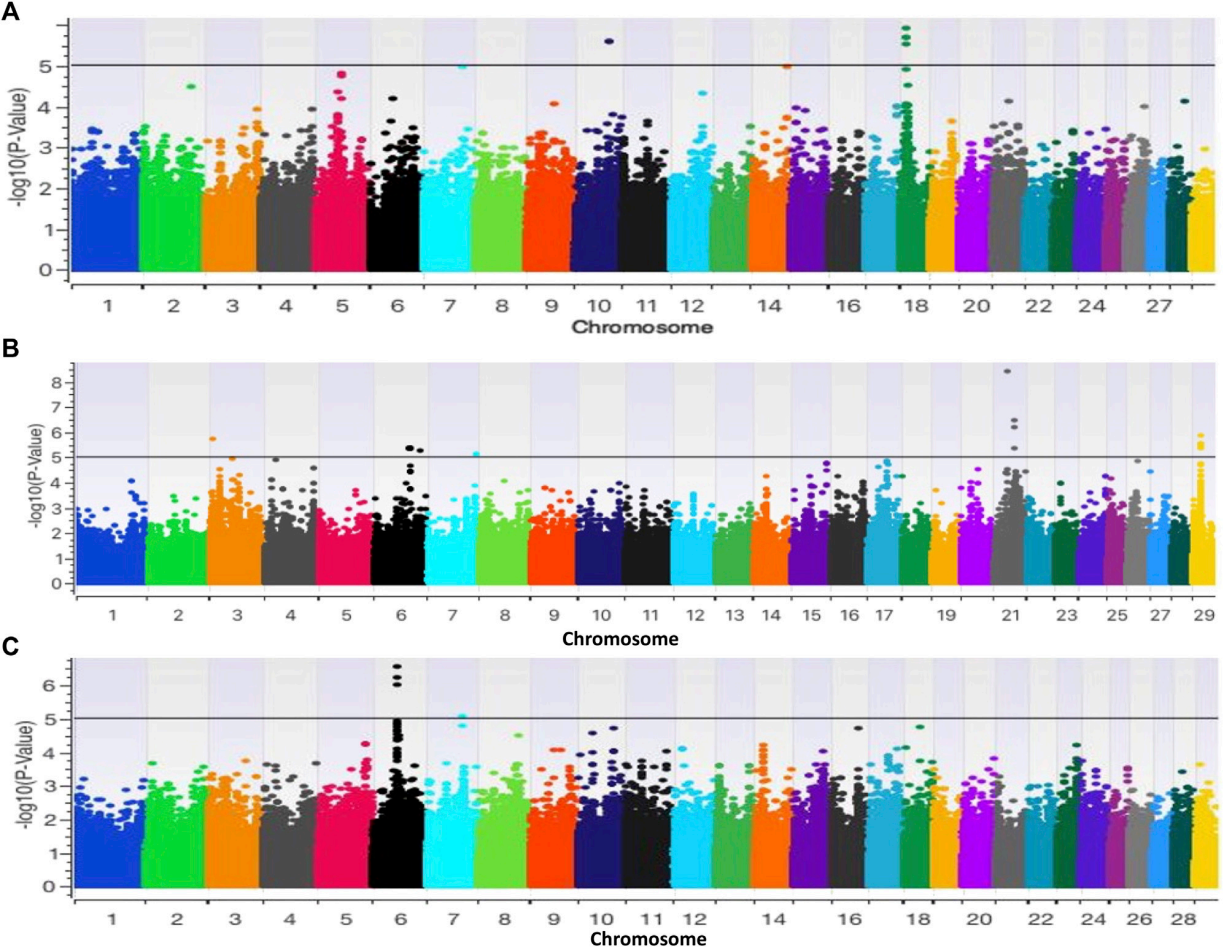
Manhattan plots based on GWAS of **(A)** base coat colour; **(B)** Forehead stripe and **(C)** coloursided phenotypes.

The GWAS for base coat colour (eumelanin vs. pheomelanin) resulted in two and four significant (p < 0.000001) SNPs on BTA10 and BTA18 respectively. (Figure 5A). The two SNPs on BTA10 (BovineHD1000021741 and BovineHD1000021742) yielded twenty-three genes, five of which had roles in 7 biological pathways that included the MAPK signalling pathway (Supplementary Table S1). The SNPs on BTA18 yielded nine genes (Supplementary Table S2). Of these genes, MYLK3 gene was found to have a role in the calcium signalling pathway, cGMP-PKG signalling pathway, vascular smooth muscle contraction, apelin signalling pathway, focal adhesion, platelet activation, regulation of actin cytoskeleton, oxytocin signalling pathway and gastric acid secretion. MC1R gene is involved in the neuroactive ligand-receptor interaction and melanogenesis pathways while the FANCA gene was observed in the Fanconi anemia pathway. Two of the identified biological pathways, the MAPK and melanogenesis pathways, are directly linked to the synthesis of coat colour (Fan et al., 2013; Xue et al., 2018).

### Colour-Sidedness and White Forehead Stripe

The GWAS for colour-sided versus non-colour-sided animals resulted in four significant SNPs on BTA6 (3 SNPs) and on BTA7 (1 SNP) as illustrated in Figure 5B. Genes such as PCDH7, LOC784827, LOC104968962, LOC104968963, LOC785099 were within 1 Mb of significant SNPs and were not associated with any pathways based on KEGG (Supplementary Table S3). The GWAS for white forehead stripe resulted in 27 indicative SNPs located on BTA3, 6, 7, 21 and 29 (Figure 5C). BTA3 harboured one indicative SNP, BTA6 harboured 16 indicative SNPs, BTA7 harboured 2 indicative SNPs while BTA21 and BTA29 harboured 4 indicative SNPs each. The indicative SNPs and genes associated with white forehead stripe are illustrated in Supplementary Table S4. The EFNA5, MAPK10, PAK1 genes were associated with the MAPK signaling pathway, while the PPP2R3C gene was found to be associated with the adrenergic signaling pathway.

Per marker pairwise F_ST_ for the different phenotypes generated similar genomic regions as GWAS analysis for forehead stripe on chromosome 6 and 21 and for colour-sidedness phenotype on chromosome 6 as illustrated in Supplementary Table S5.

## Discussion

Coat colour and patterns have been widely used to differentiate cattle breeds (Yang et al., 2013), identify and select for other socio-cultural and adaptive traits. Coat colour traits are becoming increasingly important because of the roles they play in the animals’ behavioural and adaptation attributes in different environments (Koseniuk et al., 2018). In this study, Nguni cattle from Bartlow Combine and Kokstadt research stations provided an opportunity to study the genetics of the different coat colour patterns observed on this breed. The study focused on base coat colours, colour-sidedness and the white forehead stripe in the hope to improve selection for coat colour in future breeding programmes.

The genetic variability of the Nguni cattle from Bartlow Combine and Kokstadt were similar to each other and higher that an average H_E_ of 0.28 reported in a genetic diversity study on six South African cattle breeds (Makina et al., 2014). Another study that was conducted using microsatellite markers reported a higher average value for the expected heterozygosity of 0.701 (Sanarana et al., 2016). The high heterozygosity values reported by Sanarana and others could possibly be due to longer periods of natural selection for adaptation and admixture in the studied populations (Ojango et al., 2011). Results for the genetic diversity analysis of the Bartlow Combine and Kokstadt Nguni cattle also showed that the two ecotypes had low inbreeding coefficients demonstrating that the individual animals from Bartlow Combine and Kokstadt were not related and the populations behaved like outbred populations. The Bartlow Combine conservation station was established in 1954 using cattle from the Mhlabisa and Nongoma districts. Today, collaboration between the Department of Agriculture, Forestry and Fisheries (DAFF) and the Industrial Development Corporation (IDC) allows Nguni cattle to be given to different farmers for breeding over a period of 5 years in an effort to increase the numbers of the Nguni cattle in rural communities (de Waal 2014). After the 5-year period, the same number of Nguni heifers and bulls must be returned to the herd. Similar dynamics characterise the Kokstadt herd and this continuous introduction of new stock from different sources to the research stations might explain why the two herds behaved like outbred populations with low inbreeding coefficients. The genetic relationship matrix confirmed the inbreeding coefficient results by reporting low kinship estimates within and between herds.

Population structure was analysed using principal component analysis. The two separate, but compact clusters observed within PC1 indicates that the two populations are genetically distinct from each other whilst a third genetic cluster consisting of a mixture of animals from Bartlow and Kokstadt farms demonstrates presence of admixed animals in line with the population dynamics of the sampled Nguni cattle whereby they were also purchased from various sources countrywide into the research stations where they were then disseminated into emerging farming communities (Kars 1993). The PCA results indicate that there was high genetic diversity within the Nguni cattle populations as well as low genetic diversity between the Bartlow and Kokstadt populations, which is expected of herds of such a broad founder population. Generally Nguni cattle exists as ecotypes reared in different ethnic and geographical communities and the herds at both Bartlow and Kokstadt were drawn from such diverse ecotypes. The 3 distinct clusters are therefore suggestive of the unique and overlapping ecotypes between the two herds. All three PCA and GRM analysis for the different phenotypes (base coat colour, forehead stripe and colour-sidedness) suggested weak population structure and low genetic relations between the animals belonging to the cases and those from the control groups. Based on the PCA and the GRM analysis, it was therefore assumed that the Nguni cattle used in this study were highly unrelated individuals, sampled from diverse gene pools and representative of the diversity prevalent in the breed. The GWAS analysis further assumed absence of population structure within and amongst the different phenotypes. These assumptions were further confirmed by the QQ plots and the average genomic inflation factors that both suggested little or no residual population structure effects on the test statistic.

Genome-wide association studies enable researchers to screen through the entire genome of the species of interest and identify genomic factors/regions associated with the trait of interest. GWAS has recently been used to map candidate genes that were associated with the coat colours of Iranian Markhoz goat (Nazari-Ghadikolaei et al., 2018) and the Brazilian Gir cattle (Riffert 2015), but has not yet been used to gain insight into the genetics underlying the coat colour variations of the South African Nguni cattle. The GWAS approach first identifies SNPs that are associated with the different phenotypes followed by inference of the genomic regions within the associated SNPs, an analysis that requires defining the window period for which to screen for associated genes. LD analysis of the Nguni herds used in this study, reported an average LD between 0.40 and 0.41 in the two herds in agreement with previous studies that reported an LD of 0.45 in Nguni cattle breed (Makina et al., 2015). LD decay analysis showed a sustained LD that was still above 0.1 at 1000 kb window. The results did suggest as with that of Makina et al. (2015) and others utility of the genomewide SNP panels in GWAS and QTL mapping studies.

The determination of the SNP window within which to screen for genes has varied between studies with no consensus as to the distance cutoff (Brodie et al., 2016). According to Brodie et al. (2016), one way to approach gene identification in GWAS studies is to closely link the probable genes to pathways. This is based on the assumption that SNPs that are associated with the same phenotype are expected to affect the same biological processes and hence the same pathways. We therefore, in this study used that approach of first identifying genes in close proximity and then secondly inferring on the associated phenotypes and their link to coat colour traits.

The genome-wide association analysis for base coat colour (eumelanin vs. pheomelanin) yielded indicative SNPs on BTA10 and BTA18. The SNPs on BTA10 (BovineHD1000021741 and BovineHD1000021742) had no gene within 1 Mb region known to be associated with coat colour. The well-known MC1R gene was however found in the vicinity of the indicative SNPs on BTA18. The MC1R gene harboured by chromosome 18 was first reported to be responsible for the black and brown pigmentations of cattle coat colours in 1995 (Klungland et al., 1995). In this study, three alleles (E^D^, E^+^, e) of the bovine extension (E) locus, that make the MC1R, were characterized. The MC1R gene was further reported to be associated with the dark coloured coats of Gir cattle (Riffert 2015). The observation of MC1R association with base colour in Nguni cattle in this study further supports the role of this gene in coat colour in multiple cattle breeds. The MC1R gene has been identified and reported in other species such as pigeons’ plumage (Ran et al., 2016) and the Chinese sheep coat colour variations (Yang et al., 2013). The study on pigeons plumage identified the MC1R gene to be associated with the trait of black plumage pigeons (Ran et al., 2016). The study on the Chinese sheep found haplotype H1 and H3 of the identified SNPs on the MC1R to be significantly associated with the Minxian Black-fur sheep population (Yang et al., 2013).

ASIP has been regarded as a regulatory protein for the melanin production because when it binds to the MC1R, it suppresses the formation of eumelanin and activates the production of pheomelanin (Videira et al., 2013). The ASIP gene was not detected in our base coat colour GWAS analysis probably because it is an antagonist to MC1R G-protein receptor. Another reason for the undetected ASIP gene in this study could be because there were mutations that occurred on the gene that led to production of eumelanin in the presence of functional and non-mutated genes such as the MC1R and its ligands, α-MSH and ACTH (Almathen et al., 2018). Studies that show mutations to the ASIP gene giving rise to the production of eumelanin have been reported in black Xalda sheep (Royo et al., 2008) and the silver fox (Våge et al., 1997). Another study reported that two missense and one deletion of in exon 4 identified on the ASIP gene were associated with dark coloured coats in alpaca (Feeley et al., 2011).

Two major pathways associated with base coat colour were observed 1) the MAPK and 2) melanogenesis pathways. The MAPK pathway is one of the core pathways that lead to the pigmentation of mammals (D’Mello et al., 2016). In melanosomes, MAPK activates the melanocyte-specific transcription factor Microphthalmia (MITF) that subsequently activates tyrosinase (TYR) and tyrosine-like protein 1 (TYRP1) (Wellbrock et al., 2002). Tyrosinase is important in the catalysis of the melanin precursor tyrosine to 3,4-dihydroxyphenylalanine (l-DOPA) that ultimately produces melanin after a few reactions. The PPP2R5E gene found on BTA10 in the study has role in the PI3K-Akt signalling pathway that regulates the MAPK in B16F10 mouse melanoma cells (Zhou et al., 2017). The AICAR adiponectin via the AMPK pathway has been used to regulate the production of tyrosinase that causes melisma in human and mouse melanocytes thus inactivating the MAPK pathway (Bang et al., 2017). A study conducted on humans showed that pigment cells that were treated with oestrogen had increased cAMP levels that ultimately activated the tyrosinase enzymes to produce eumelanin and cells treated with progesterone had an opposite effect (Natale et al., 2016). The HSPA2 gene found on BTA10 was also involved in the MAPK pathway. The MC1R gene identified on BTA18 in the GWAS results for base coat colours is associated with the core pathway that is responsible for melanogenesis. This gene is G-protein coupled receptor found on the surface of melanin-producing cells that binds α-MSH/ACTH/ASIP thus activating a series of signalling pathways in the cytoplasm of melanocytes (Millington 2006).

In our study, the KIT gene was surprisingly not observed in the vicinity of the indicative SNPs for colour-sided Nguni cattle. According to (Olson 1999), the gene for colour-sidedness (*Cs*), is inherited in its dominant state and the expression of the gene in the heterozygous form brings about variations in those animals. Colour-sidedness trait has been observed in Belgian Blue and Brown Swiss cattle (Durkin et al., 2012). Durkin et al. (2012) reported the colours-sidedness trait in cattle to represent a phenomenon that results from copy number variants (CNVs) particularly duplication and translocation of a segment that harbours the KIT gene from chromosome 6 to chromosome 29 (Durkin et al., 2012). Another study conducted on colour-sided African Nguni cattle reported similar results to Durkin et al. (2012) with two normal and ectopic signals on BTA6 and BTA29, respectively (Szczerbal et al., 2017). A recent study stated that the KIT gene showed significant association to white coat coloured Iranian Markhoz goat (Nazari-Ghadikolaei et al., 2018) and another study reported that mutations experienced by this gene results in white coat colours in many species including cattle (Hanna et al., 2014). SNPs on BTA6 and SNPs within the KIT locus in this study were further than 1 Mb ruling out the possibility of the KIT gene having an influence on colour-sidedness in Nguni cattle. Our results were however similar to another study conducted on colour-sided cattle that could not find significant associations between KIT linked SNPs on BTA6 and colour-sided phenotypes. Mastrangelo et al. (2019), for example reported PLK2 on BTA20 as a potential candidate gene in colour sidedness phenotype in Cinisara cattle.

Besides the possibility of other genetic mechanisms other than those impacted by KIT gene, confounding known and unknown population structures implicate GWAS results and when not accounted for may lead to false positives and false negatives (Zhang and Pan 2015). Also, phenotypic complexities can interfere with determining the SNPs associated with the trait of interest on our GWAS analysis. The Nguni coat colour patterns are complex, which made phenotyping for colour sidedness with precision and objectivity challenging in this study. This included the existence of multiple phenotypes in a single animal i.e., colour-sidedness and forehead stripes and other phenotypes not reported in this study. It is therefore recommended that more comprehensive phenotyping, with a larger sample size and diverse colour sided patterns is conducted before concluding on the genetics of colour sidedness in this breed.

When melanogenesis is disrupted, leucism can occur. Leucism is a condition where there is lack of pigmentation due to the absence of melanocytes on the animals’ skin and/or coat colours and results in what is seen as white spots or patches on a solid base colour. These phenotypes include the white forehead stripe on the cattle’s heads and white patterns observed on the animals’ body and head. White phenotypes can also be desirable traits to some farmers or breeders in hot humid environments because animals can tolerate heat and function at their optimal. Nguni cattle have been reported to be an optimal breed for the production of beef and have immunity against diseases (Mapholi et al., 2014). It is thus believed that the diversity of coat colours and myriad of coat colour patterns have played a role in some of its adaptive traits. The GWAS conducted for the white forehead stripe resulted in indicative SNPs on BTA6, 7, 21 and 29. The KIT gene was not found in the vicinity of the indicative SNPs. However, the four genes MAPK10, EFNA5, PPP2R3C and PAK1 harboured on BTA6, 7, 21 and 29, respectively were found to be closely associated with the melanogenesis. The MAPK10, EFNA5 and PAK1 are associated with the MAPK signaling pathway while MAPK10 is also found to be associated with the Wnt signaling pathway. The Wnt signaling pathway plays a critical role in the developmental and differentiation stages of melanocytes (D’Mello et al., 2016). Wnt1 and Wnt3a are responsible for the increment of melanocytes and differentiation of neural crest cells to melanocytes in the presence of beta-catenin, respectively (Dunn et al., 2005). When beta-catenin is bound to the Wnt proteins, it regulates MITF transcription via LEF/TCF transcription factors (Steingrímsson et al., 2004). The MITF gene is activated through the MAPK pathway and this gene is responsible for the regulation of tyrosinase (TYR) that activates melanogenesis in melanocytes. The PPP2R3C gene is associated with the adrenergic signaling pathway, which is also important in melanogenesis pathway. When the norepinephrine is bound to the adrenergic receptors, the inositol triphosphate/diacylgycerol that controls the release of calcium is activated (Park et al., 2009). Subsequently, the PKC-beta is activated thus activating TYR by phosphorylation in cells to commence with melanin synthesis. The TECRL gene has been previously found to have associations with pigmentation patterns of the Fleckvieh cattle’s head (O’brien et al., 2014). Literature reports that the TECRL gene also plays a role in lipid chemical reactions and signaling pathways (O’brien et al., 2014).

Overall the GWAS on presence of white forehead stripe in Nguni cattle demonstrated role of different genes at play that impact pathways known to have an influence on melanogenesis and therefore pigmentation in vertebrates. This could imply either genetic heterogeneity of coat colour and patterns phenotype or complexity of coat colour phenotypes in Nguni cattle leading to confounding effects. A more comprehensive study with intensive phenotyping of white forehead stripe is therefore required.

## Conclusion

Overall, the GWAS for coat colour patterns in the South African Nguni cattle shed light into the genetics of coat colour patterns in this breed. The GWAS for base coat colour reported SNPs, genes and pathways similar to those from other studies, with the observation of MC1R gene, which is well known to be associated with coat colour in cattle and other species. This gene was also observed to be linked to the well-known MAPK and melanogenesis pathways. Although, the GWAS for colour-sidedness and white forehead stripe did not report the well-known KIT gene, the significant genes such as MAPK10, EFNA5, PPP2R3C and PAK1 are synergistically involved in the synthesis of melanin. Another gene, TECRL, on BTA6 observed to be associated with white forehead stripe phenotype in this study has been reported to be associated with pigmentation patterns in Fleckvieh cattle. Because of the complexity of coat colour patterns in the Nguni cattle, this study recommends comprehensive sampling and phenotyping particularly of leucism traits for future GWAS studies.

## Data Availability

The data analyzed in this study is subject to the following licenses/restrictions: The datasets generated and/or analyzed during the current study are available from the OSF repository using the link: https://osf.io/t36bw/.
