# Supplementary figures and images for: Genetics of Base Coat Colour Variations and Coat Colour-Patterns of the South African Nguni Cattle Investigated Using High-Density SNP Genotypes
Source: Front Genet. 2022 Jun 7;13:832702. doi: 10.3389/fgene.2022.832702 (PMC9209731; doi:10.3389/fgene.2022.832702)

(a)

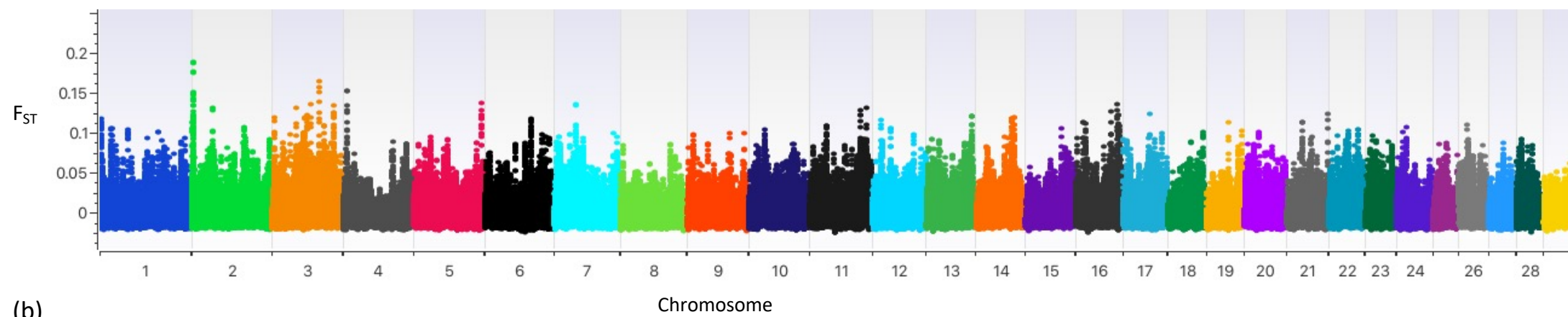

(b)

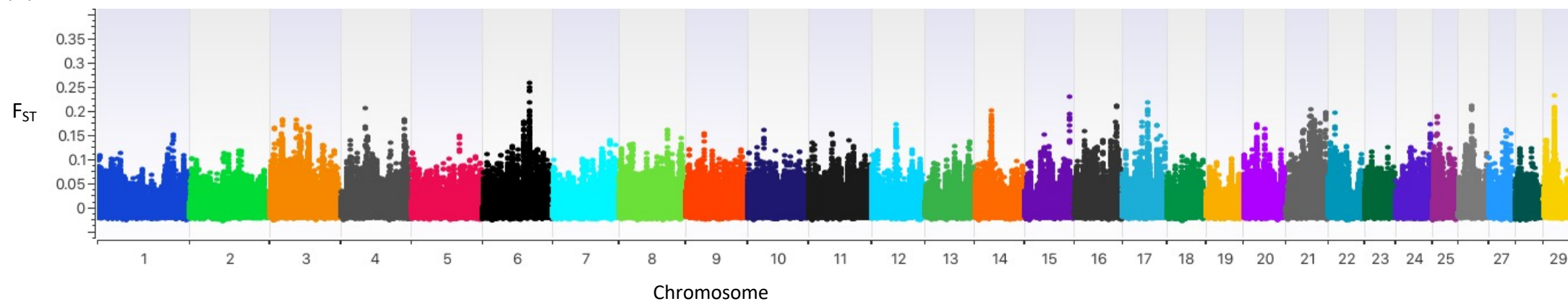

(c)

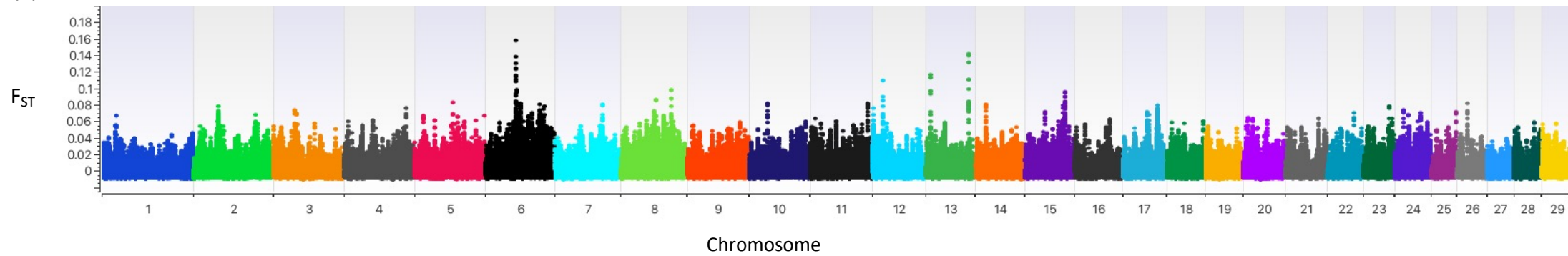

Supplement: Supplementary file 1 [file Table5.pdf]

|            | Bartlow  | Kokstadt |
|------------|----------|----------|
| 0-1 kb     | 0,5936   | 0,6067   |
| 1-2 kb     | 0,5465   | 0,5602   |
| 2-3 kb     | 0,4928   | 0,5068   |
| 3-4 kb     | 0,45     | 0,46365  |
| 4-5 kb     | 0,42895  | 0,44258  |
| 5-6 kb     | 0,40808  | 0,42253  |
| 6-7 kb     | 0,39334  | 0,40702  |
| 7-8 kb     | 0,3783   | 0,39266  |
| 8-9 kb     | 0,3653   | 0,37862  |
| 9-10 kb    | 0,35333  | 0,36705  |
| 10-20 kb   | 0,30526  | 0,31801  |
| 20-30 kb   | 0,25545  | 0,26788  |
| 30-40 kb   | 0,23657  | 0,24915  |
| 40-50 kb   | 0,22751  | 0,23896  |
| 50-60 kb   | 0,22125  | 0,23213  |
| 60-70 kb   | 0,21785  | 0,22818  |
| 70-80 kb   | 0,21721  | 0,2245   |
| 80-90 kb   | 0,21406  | 0,22222  |
| 90-100 kb  | 0,20841  | 0,2204   |
| 100-200 kt | 0,2011   | 0,21502  |
| 200-300 kt | 0,17876  | 0,1938   |
| 300-400 kt | 0,14225  | 0,17609  |
| 400-500 kt | 0,168227 | 0,18507  |
| 500-600 kt | 0,165174 | 0,16714  |
| 600-700 kt | 0,20281  | 0,16979  |
| 700-800 kt | 0,154207 | 0,150284 |
| 800-900 kt | 0,109088 | 0,12336  |
| 900-1000k  | 0,088611 | 0,114207 |
| 1000-2000  | 0,05001  | 0,09927  |

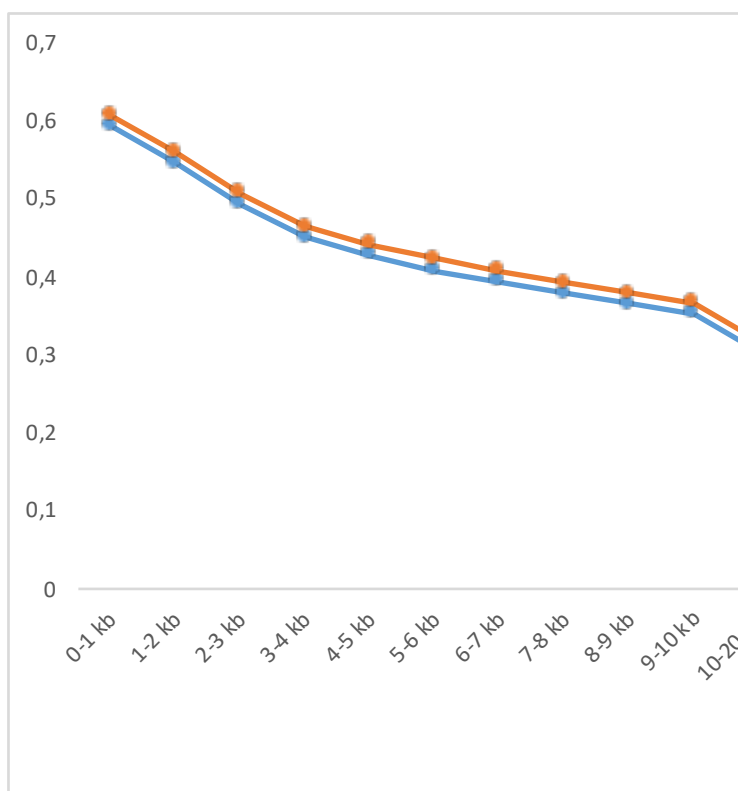

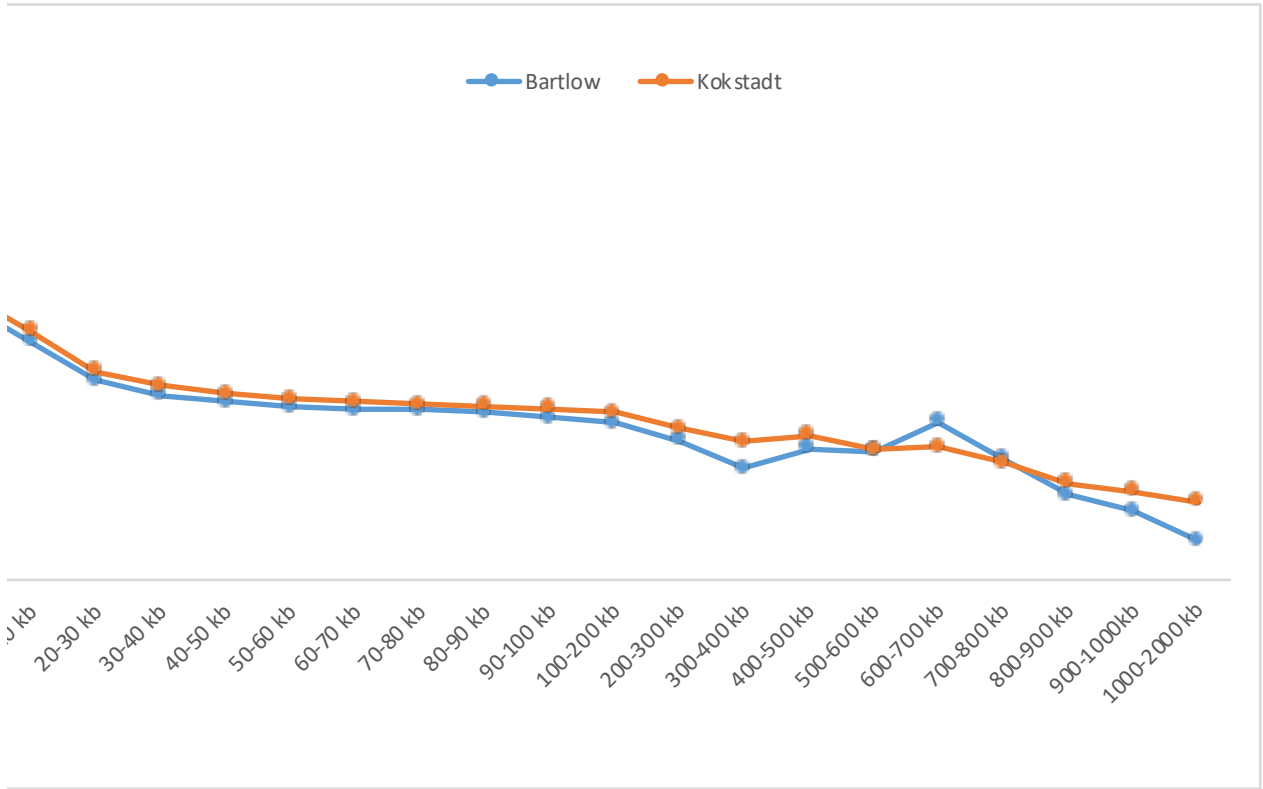

Supplement: Supplementary file 4 [file DataSheet1.PDF]
