# Supplementary material for: Genetics of Base Coat Colour Variations and Coat Colour-Patterns of the South African Nguni Cattle Investigated Using High-Density SNP Genotypes
Source: Front Genet. 2022 Jun 7;13:832702. doi: 10.3389/fgene.2022.832702 (PMC9209731; doi:10.3389/fgene.2022.832702)
Supplement: Supplementary file 3 [file Table2.docx]

**Supplementary Table S****2 Indicative SNPs on BTA10 and BTA18 for the base coat colours in Nguni cattle and the candidate genes and their associated signalling pathways**

| BTA | SNP Name | SNP Position | Candidate genes | KEGG Pathway |
| --- | --- | --- | --- | --- |
| 10 | BovineHD1000021741 | 76313122 | *LOC107132878, KCNH5, RHOJ,*  *GPHB5, LOC783427, LOC783478, PPP2R5E, WDR89,*  *LOC101904356, SGPP1,*  *LOC104973205, LOC107132885,*  *LOC104973248, SYNE2,*  *LOC100850347, LOC101904397,*  *ESR2, LOC104973206,*  *LOC100297513,*  *MTHFD1, AKAP5, ZBTB25,*  *ZBTB1, HSPA2, PPP1R36.* | *PPP2R5E*- mRNA surveillance pathway, Sphingolipid signalling Oocyte meiosis, PI3K-Akt signalling pathway, AMPK signalling pathway, Adrenergic signalling in cardiomyocytes, Dopaminergic synapse, Human papillomavirus infection |
|  | BovineHD1000021742 | 76314115 |  | *SGPP1*-Sphingolipid signalling pathway, Sphingolipid metabolism  *ESR2*- Estrogen signalling pathway, GnRH secretion, Pathways in cancer, Prolactin signalling pathway, Breast cancer, Endocrine resistance  *HSPA2*- Estrogen signalling pathway, Legionellosis, MAPK signalling pathway, Antigen processing and presentation, Endocytosis, Spliceosome, Longevity regulating pathway, Protein processing in endoplasmic reticulum, Toxoplasmosis, Measles  *MTHFD1*- One carbon pool by folate, Metabolic pathways |
| 18 | ARS-BFGL-NGS-10007 | 15483066 | *ANKRD11, SPG7, RPL13, CPNE7, DPEP1, CHMP1A,*  *SPATA33, LOC104974758,*  *CDK10, SPATA2L, VPS9D1,*  *VPS9D1, ZNF276, FANCA, SPIRE2,*  *LOC107131144, TCF25,*  *MC1R, TUBB3, DEF8, CENPBD1, LOC532875,*  *DBNDD1, GAS8,*  *LOC100296324,*  *LOC104974835, SHCBP1,*  *LOC101904595, VPS35,*  *ORC6, MYLK3, LOC104974760,*  *C18H16orf87, LOC101905644,*  *LOC104974761, GPT2, DNAJA2,*  *LOC789587, LOC100847949,*  *NETO2, ITFG1, PHKB,*  *LOC104974762, LOC533093.* | *MYLK3*- Calcium signalling pathway, cGMP-PKG signalling pathway, Vascular smooth muscle contraction, Apelin signalling pathway, Focal adhesion, Platelet activation, Regulation of actin cytoskeleton, Oxytocin signalling pathway, Gastric acid secretion  *TUBB3*- Phagosome, Gap junction |
|  | BovineHD1800004871 | 15484331 |  | *GPT2*- Arginine biosynthesis, Alanine, aspartate and glutamate metabolism, Metabolic pathways, Carbon metabolism, 2-Oxocarboxylic acid metabolism, Biosynthesis of amino acids |
|  | BovineHD1800004872 | 15486496 |  | *RPL13*- Ribosome |
|  | BovineHD1800004878 | 15492044 |  | *SPATA2L*- Necroptosis  *ORC6*- Cell cycle  *DNAJA2*- Protein processing in endoplasmic reticulum  *MC1R*- Neuroactive ligand-receptor interaction, Melanogenesis  *FANCA*- Fanconi anemia pathway |
