# Supplementary material for: Genetics of Base Coat Colour Variations and Coat Colour-Patterns of the South African Nguni Cattle Investigated Using High-Density SNP Genotypes
Source: Front Genet. 2022 Jun 7;13:832702. doi: 10.3389/fgene.2022.832702 (PMC9209731; doi:10.3389/fgene.2022.832702)
Supplement: Supplementary file 5 [file Table3.docx]

**Supplementary Table S****3 Indicative SNPs and candidate genes found on BTA6 and BTA7 for the colour-sidedness trait on Nguni cattle**

| BTA | SNP Name | SNP Position | Candidate genes | KEGG Pathway |
| --- | --- | --- | --- | --- |
| 6 | BovineHD0600014333 | 51960004 | *PCDH7, LOC784827* | No significant pathway associated with identified genes |
|  | BTB-01345846 | 51964857 |  |  |
|  | BovineHD0600014334 | 51967625 |  |  |
| 7 | BovineHD0700023152 | 79094579 | *LOC104968962*  *LOC104968963*  *LOC785099* | No significant pathway associated with identified genes |
