# Supplementary material for: Genetics of Base Coat Colour Variations and Coat Colour-Patterns of the South African Nguni Cattle Investigated Using High-Density SNP Genotypes
Source: Front Genet. 2022 Jun 7;13:832702. doi: 10.3389/fgene.2022.832702 (PMC9209731; doi:10.3389/fgene.2022.832702)
Supplement: Supplementary file 6 [file Table4.docx]

Supplementray Table S4 **Indicative SNPs and the number of genes for the white forehead stripe trait on Nguni cattle**

| CHR | SNP Name | SNP Position | Genes | KEGG Pathway |
| --- | --- | --- | --- | --- |
| 3 | BovineHD0600035925 | 6687516 | 21 | None |
| 6 | BovineHD0600022092 | 80050684 | 4 |  |
|  | BovineHD0600022094 | 80071203 |  |  |
|  | BovineHD0600022159 | 80338390 | 5 |  |
|  | BovineHD0600022167 | 80411239 |  |  |
|  | BovineHD0600022168 | 80416015 |  |  |
|  | BovineHD0600022169 | 80424155 |  |  |
|  | BovineHD0600034789 | 80447570 |  |  |
|  | BovineHD0600022189 | 80515667 |  |  |
|  | BovineHD0600022190 | 80521617 |  |  |
|  | BovineHD0600022193 | 80550852 | 6 |  |
|  | BovineHD0600022199 | 80587196 |  |  |
|  | BovineHD0600022200 | 80594600 |  |  |
|  | BovineHD0600022285 | 80864147 | 5 including  *TECRL* |  |
|  | BovineHD0600022286 | 80869971 |  |  |
|  | BovineHD0600022312 | 80997999 |  |  |
|  | BTA-14100-rs29022867 | 103056415 | 10 including  *MAPK10* | MAPK signalling pathway |
| 7 | BovineHD0700032090 | 109855791 | 9 including *EFNA5* | MAPK signalling pathway |
|  | ARS-BFGL-NGS-81660 | 109890025 |  |  |
| 21 | BovineHD2100008758 | 30428573 | 32 |  |
|  | BovineHD2100013388 | 46767216 | 30 including *PPP2R3C* | Adrenergic Signalling pathway |
|  | BovineHD2100013397 | 46784435 |  |  |
|  | BovineHD2100013400 | 46791691 |  |  |
| 29 | BovineHD2900005672 | 19661149 | 14 including *PAK1* | MAPK signalling pathway |
|  | BovineHD2900005703 | 19758698 |  |  |
|  | BovineHD2900005705 | 19763844 |  |  |
|  | BovineHD2900005718 | 19797933 |  |  |
